# The genetic architecture of aniridia and Gillespie syndrome

**DOI:** 10.1007/s00439-018-1934-8

**Published:** 2018-09-22

**Authors:** Hildegard Nikki Hall, Kathleen A. Williamson, David R. FitzPatrick

**Affiliations:** MRC Human Genetics Unit, MRC Institute of Genetics and Molecular Medicine, The University of Edinburgh, Western General Hospital, Crewe Road, Edinburgh, EH4 2XU UK

## Abstract

Absence of part or all of the iris, aniridia, is a feature of several genetically distinct conditions. This review focuses on iris development and then the clinical features and molecular genetics of these iris malformations. Classical aniridia, a panocular eye malformation including foveal hypoplasia, is the archetypal phenotype associated with heterozygous *PAX6* loss-of-function mutations. Since this was identified in 1991, many genetic mechanisms of *PAX6* inactivation have been elucidated, the commonest alleles being intragenic mutations causing premature stop codons, followed by those causing C-terminal extensions. Rarely, aniridia cases are associated with *FOXC1, PITX2* and/or their regulatory regions. Aniridia can also occur as a component of many severe global eye malformations. Gillespie syndrome—a triad of partial aniridia, non-progressive cerebellar ataxia and intellectual disability—is phenotypically and genotypically distinct from classical aniridia. The causative gene has recently been identified as *ITPR1*. The same characteristic Gillespie syndrome-like iris, with aplasia of the pupillary sphincter and a scalloped margin, is seen in *ACTA2*-related multisystemic smooth muscle dysfunction syndrome. WAGR syndrome (Wilms tumour, aniridia, genitourinary anomalies and mental retardation/intellectual disability), is caused by contiguous deletion of *PAX6* and *WT1* on chromosome 11p. Deletions encompassing *BDNF* have been causally implicated in the obesity and intellectual disability associated with the condition. Lastly, we outline a genetic investigation strategy for aniridia in light of recent developments, suggesting an approach based principally on chromosomal array and gene panel testing. This strategy aims to test all known aniridia loci—including the rarer, life-limiting causes—whilst remaining simple and practical.

## Structure and development of the human iris

Sitting between the cornea and lens of the eye, the adult human iris is a 12 mm vivid, multilayered and photoprotective disc. Through control of pupil size in response to light or accommodation, it regulates light passage to the retina and is an optical requirement for sharp vision. It lies anterior to the ciliary body and itself plays a structurally important role in aqueous dynamics and the iridocorneal angle configuration, both of which affect intraocular pressure and glaucoma risk.

**Fig. 1:**
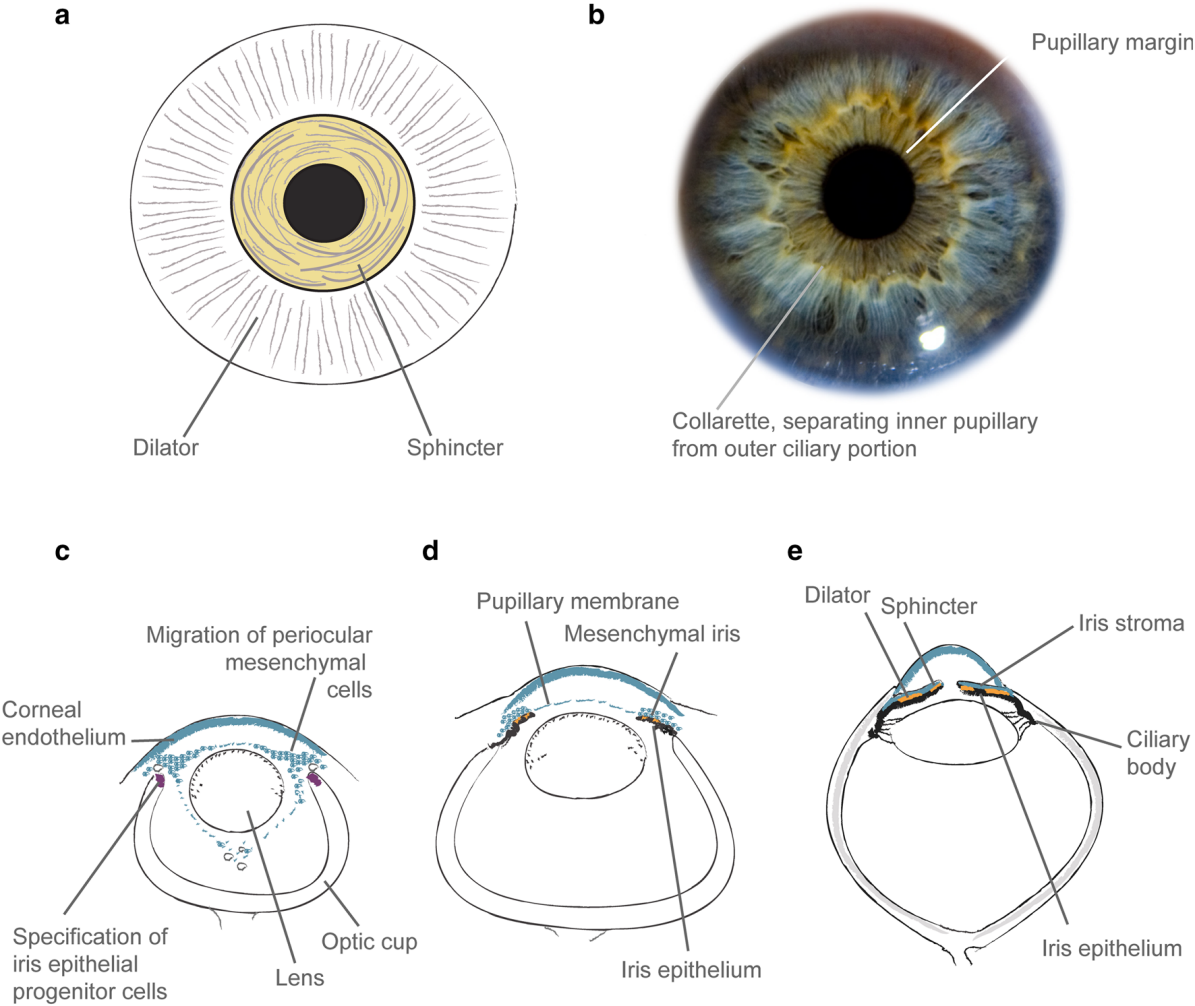
The structure and development of the human iris. **a** Cartoon of the iris musculature, showing the position of the sphincter (shaded yellow) and dilator muscles. **b** Macroscopic photo of normal adult iris (courtesy of Chris Moody), showing the inner pupillary portion and outer ciliary portion. **c** Cartoon of the developing eye in a 7–8-week-old human embryo, showing the migration of periocular mesenchymal cells (shaded in teal) into the developing anterior chamber, which will go on to form the mesenchymal iris, pupillary membrane and corneal endothelium and stroma. The tips of the optic cup are shaded in purple showing the specification of iris epithelial progenitor cells. **d** Cartoon of developing human eye at 4–5 months’ gestation, showing the iris growing out from the optic cup margins. The iris musculature (shaded orange) is just starting to develop. **e** Cartoon of adult eye, showing a well-formed anterior chamber and iridocorneal angle; the iris stroma, musculature (orange) and epithelium (black); lens attached by zonules to the ciliary muscle. The cartoons in **a, b** are derived from multiple sources (primarily Mann 1925)

A macroscopic view of the iris shows a central pupillary region demarcated from the ciliary region by the iris collarette (Fig. 1). The sphincter muscle, responsible for miosis (constriction) under parasympathetic nervous control, lies in this inner pupillary region, whereas the dilator muscle, which produces sympathetically mediated mydriasis, lies in the more peripheral ciliary region.

Of the principal components of the iris, the bilayered epithelium and smooth muscle have a different embryological origin to the overlying stroma.

The iris pigmented epithelium is neuroectodermal, developing as an outgrowth from the margins of the optic cup from the third month of gestation—relatively late compared to the rest of the eye (Fig. 1). Specification of iris progenitor cells to a non-neuronal fate is accompanied by the expression of transcription factors including PAX6 and OTX1, and of members of the Wnt signalling pathway (Liu et al. 2007; reviewed by Davis-Silberman and Ashery-Padan 2008). Notch genes are also implicated in this patterning (Bao and Cepko 1997). Recently, two members of the TGFβ superfamily (*Bmp4* and *Tgfβ2*) and *Foxc1* have been identified as direct downstream targets of Pax6 in murine iris development (Wang et al. 2016). In addition to evidence that they are expressed in mouse iris development (Chang et al. 2001; Adams et al. 2007; Hägglund et al. 2017), *BMP4* and *BMP7* are highly expressed in developing chick iris smooth muscle (Jensen 2005). The iris sphincter and dilator muscles are rare examples of ectodermally derived muscle (Imaizumi and Kuwabara 1971; Jensen 2005). They are not fully formed histologically in human fetuses until the sixth and eight months, respectively (Mann 1925).

By contrast, the pupillary membrane and iris stroma are mesenchymal in origin, arising from the third wave of migrating neural crest cells (Williams and Bohnsack 2015). During the second month of gestation, after separation of the lens but before the appearance of iris epithelium, a thin layer of mesodermally derived tissue is visible anterior to the lens (Mann 1925). The central part of this will become the pupillary membrane, which regresses late in gestation. Mesenchymal cells migrate along the anterior border of the iris epithelium, along with stromal melanocytes also deriving from neural crest (Sturm and Larsson 2009). Retinoic acid and its downstream target *PITX2* are critical to regulating neural crest derivatives in the anterior segment (Williams and Bohnsack 2015). *PAX6* is also expressed during morphogenesis of this part of the iris, though in a more transitory manner and at lower levels than in the ectodermal tissues (Cvekl and Tamm 2004). The *Small eye* rat (*rSey*) shows impaired migration of neural crest cells from the future anterior midbrain to the eye rudiment (Matsuo et al. 1993).

The striking visible colour of the iris is directly related to both the concentration of pigment within iris stromal melanocytes, and the type of melanin (eumelanin:pheomelanin ratio) (Prota et al. 1998). Final adult eye colour is not reached at birth, with iris stromal development continuing postnatally. In Caucasian infants, the iris is bluer for the initial months of life due to lower stromal melanin content (Rennie 2012). Genetically, the *OCA2-HERC2* region of chromosome 15q explains the large majority of variation in human eye colour (Sturm and Larsson 2009).

## Classical aniridia

### Clinical features

**Fig. 2:**
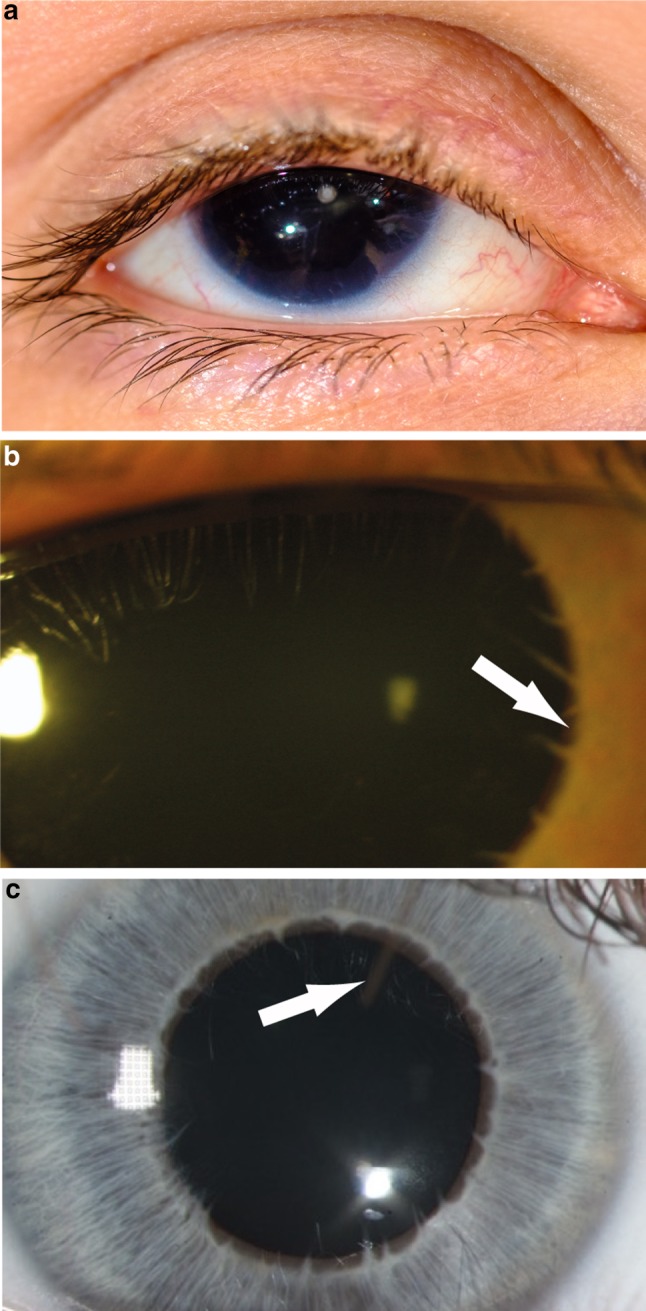
Iris phenotypes in classical aniridia, Gillespie syndrome and multisystemic smooth muscle dysfunction syndrome. **a** Right eye of an individual with classical aniridia, showing near complete absence of the iris and mild ptosis (image courtesy of David Hall); **b** iris of a Gillespie syndrome patient with an *ITPR1* mutation. Note the scalloped edge of the iris remnant (arrow), with aplasia of the iris central to the collarette (image courtesy of Abhijit Dixit); **c** iris of a patient with an *ACTA2* mutation and multisystemic smooth muscle dysfunction syndrome, showing the aplasia of the iris central to the collarette, with a scalloped pupillary margin and iridolenticular strand (arrow) (image courtesy of Françoise Meire)

First described in 1818 (Barratta 1818), classical aniridia is a dominantly inherited, usually bilateral, panocular malformation (OMIM 106210). Two comprehensive Scandinavian studies have shown a prevalence of between 1 in 40,000 and 72,000 live births (Grønskov et al. 2001; Eden et al. 2008), in keeping with older US epidemiological data (Shaw et al. 1960). The term aniridia can refer to both the clinical sign and the condition (Table 1). The latter is a spectrum comprising aniridia with foveal plus or minus optic nerve hypoplasia, with later development of cataracts, glaucoma and/or keratopathy. Ptosis is also sometimes seen as a feature, as in Fig. 2.

**Table 1:** Definition of terms used in the description of aniridia and related eye malformations

Term	Working definition
Iris	
Aniridia	1. (Clinical sign) Absence of the iris
	2. (Disease) Panocular eye malformation
Complete aniridia	Absence of the iris, with no visible iris tissue (distinction is not made depending on whether any remnant is detectable goniscopically)
Partial aniridia	Incomplete aniridia with some visible iris rim (e.g., in classical aniridia) or, less commonly, absence of part of the iris (e.g., in Gillespie syndrome; this is a distinct phenotype to classical aniridia consisting of aplasia of the sphincter)
Iris hypoplasia	Unlike aniridia, this is a neutral term which does not imply any particular associated condition. In the context of aniridia, used to describe the minor end of the spectrum of hypoplasia of the iris (cf partial to complete aniridia). The mildest sign of this is iris transillumination
Congenital mydriasis	Fixed dilated pupils. In Gillespie syndrome this is due to aplasia of the sphincter
Iris coloboma	Approximately 6 o’clock defect in the iris resulting from failure of optic fissure closure
Iridolenticular strands	Strands of tissue between the iris and the lens, often seen in Gillespie syndrome or anterior segment dysgeneses
Corectopia	Displacement of the pupil, which may be misshapen
Other anterior segment	
Posterior embryotoxon	An abnormality of the iridocorneal angle resulting in an anteriorly displaced Schwalbe’s line. Characteristic of Axenfeld–Rieger syndrome but seen in 15% normal eyes
Keratopathy	A general term for corneal pathology. In the context of aniridia, this is progressive opacification of the cornea over many years, related to limbal stem cell failure
Congenital corneal opacification	Congenital corneal opacification may result from aniridia and from other developmental anomalies of the anterior segment such as failure of lens separation. Terms such as Peters anomaly and sclerocornea are clinical signs and are not helpful aetiologically. The authors recommend the classification outlined by Nischal (2015)
Cataract	Congenital or acquired lens opacity, with the visual prognosis and need for surgery depending on the age of onset, symptoms, morphological type and laterality
Posterior segment	
Foveal hypoplasia	Failure of the specialisation of the centre of the macula for fine detailed central vision, visible on OCT as an absent or shallow foveal dip. A core feature of aniridia (but not Gillespie syndrome) and ocular albinism
Optic nerve hypoplasia	Visible fundoscopically as a small optic disc, classically with a peripapillary halo and altered ring of pigmentation, with reduced vision correlating not with disc size but with papillomacular nerve fibre integrity
Optic nerve coloboma	Excavated optic disc anomaly due to defective optic fissure closure and thus deeper inferiorly, which may involve the whole disc and/or coexist with chorioretinal coloboma and microphthalmia
Other	
Ptosis	Drooping of the upper eyelid

*OCT* optical coherence tomography

The typical iris phenotype is of complete (Fig. 2) or partial aniridia, often with a thin crescent of iris visible. Lesser degrees of iris hypoplasia are also seen. The distinction between partial and total aniridia is of limited use as, armed with a gonioscopy lens to view the iridocorneal angle, even those with apparently complete aniridia tend to have a detectable iris stump (Nelson et al. 1984). However, cases of apparent partial aniridia with aplasia of the sphincter and a scalloped margin are important to note diagnostically (see “Gillespie syndrome” section below).

Whilst congenital glaucoma presenting with buphthalmos is rare in aniridia, glaucoma occurs in approximately half of aniridia patients, in later childhood or in adulthood (Grant and Walton 1974). Mechanisms of glaucoma described in aniridia include open angle glaucoma, rotation of the iris stump causing synechial chronic angle closure, and goniodysgenesis (Netland 2015). Furthermore, some patients undergo childhood cataract surgery which itself carries an independent risk of secondary glaucoma (Mataftsi 2016). The whole spectrum of cataract morphology is seen in aniridia, but they are often polar and associated with capsular fragility that render cataract surgery more difficult (Schneider et al. 2003). Lens subluxation also occurs.

Interestingly, foveal hypoplasia in aniridia occurs without the chiasmal misrouting seen in albinism (Neveu et al. 2005). Foveal hypoplasia usually results in reduced vision, at the level of at least 6/30 (0.7 logMAR), such that most patients are eligible for sight impairment registration. The optical consequences of lack of iris tissue also contribute to this, and strabismic amblyopia (lazy eye) is frequently seen.

Lastly, abnormal corneal epithelial homeostasis and limbal stem cell deficiency in aniridia lead to progressive keratopathy (Douvaras et al. 2013). Signs of aniridic keratopathy are typically apparent from early adulthood, the cornea becoming slowly more cloudy and vascularised. This constitutes the main cause of progressive visual loss in later life (Mayer et al. 2003). Rarely, a congenital corneal opacity may be present due to keratolenticular apposition (Nischal 2015).

Whilst other ocular phenotypes such as microphthalmia are seen in conjunction with *PAX6* mutations (discussed below), this is not considered part of the classical aniridia phenotype. Non-ocular phenotypes, in particular neuroanatomical and endocrine abnormalities (both potentially asymptomatic) have been identified in studies of aniridia patients. Impaired glucose tolerance has been reported in aniridia (Yasuda et al. 2002), and the prevalence of diabetes may be slightly higher [7% vs 4.5%, though note that the populations of these studies are not directly comparable (Netland et al. 2011; Menke et al. 2015)]. This is supported by functional and animal work on the role of Pax6 in the pancreas (Swisa et al. 2017). Subtle deficits in olfaction and hearing have been described (Sisodiya et al. 2001; Bamiou et al. 2007). MRI brain studies have shown structural abnormalities on MRI in the pineal gland, anterior commissure, corpus callosum and frontoparietal cortical areas (Mitchell et al. 2003; Free et al. 2003; Yogarajah et al. 2016). These are primarily radiological studies with no, or limited (Yogarajah et al. 2016), evidence of potential functional consequences. However, there is evidence of auditory interhemispheric transfer deficits in children with aniridia (Bamiou et al. 2007).

### Identification of *PAX6*

Classical aniridia is strongly associated with heterozygous loss-of-function of *PAX6* (*Paired box 6*, OMIM 607108, hg38 chr11:31784792–31811353). Animal models and human patients were key to the identification of this gene in the early 1990s (Hanson et al. 1994). The 25th anniversary was recently commemorated in a review (Cvekl and Callaerts 2017).

Comparative mapping data had suggested that human aniridia was homologous to the murine *Sey* phenotype (van der Meer-de Jong et al. 1990; Glaser et al. 1990), a strain first reported in 1967 as having microphthalmia in heterozygotes (Roberts 1967)—and later shown to also feature iris hypoplasia, cataracts and corneal opacification (Jordan et al. 1992). *Pax6* was isolated along with other *Pax* genes from mouse expression libraries (Walther et al. 1991). In parallel, the gene was independently identified from positional cloning and shown to be deleted in human aniridia (Ton et al. 1991). Concurrent analysis of *Sey* alleles confirmed the causal link between *Pax6* and semidominant *Sey* (Hill et al. 1991).

Establishing the link between human *PAX6* mutations and the semidominant *Sey*, the authors (Hill et al. 1991) first observed the importance of dosage sensitivity. More recently, this property has been quantified with a haploinsufficiency metric by the Exome Aggregation Consortium (ExAC) (Lek et al. 2016), with an impressive probability of loss-of-function intolerance score, pLI, of 1.00 for *PAX6*. Whilst *PAX6* haploinsufficiency leads to aniridia, whole-gene duplication causes non-aniridic eye malformations, and homozygous loss-of-function is perinatally lethal in humans and mice (Roberts 1967; Glaser et al. 1994; Schedl et al. 1996; Aalfs et al. 1997; Schmidt-Sidor et al. 2009; Schilter et al. 2013).

### PAX6 biology and structure

PAX6 is a highly conserved transcription factor critical to the control of both ocular and neural development (Prosser and van Heyningen 1998). In addition to orchestrating the developing eye, it is expressed in the central nervous system and pancreas, as well as in adult tissues such as the cornea, brain and pancreas where it is involved in homeostasis (Sivak et al. 2003; Hart et al. 2013; Yogarajah et al. 2016; Huettl et al. 2016; Maurya and Mishra 2017).

**Fig. 3:**
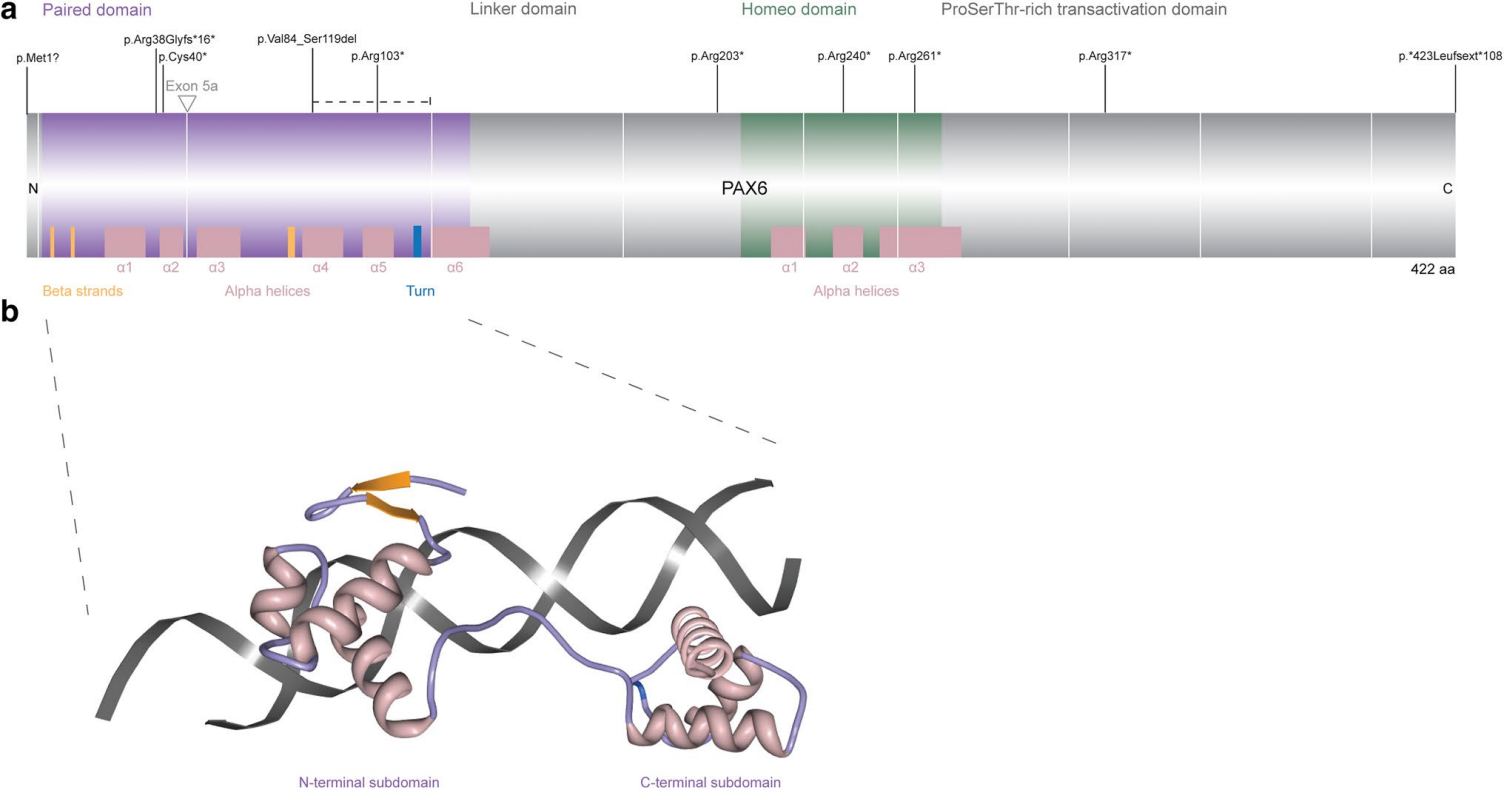
**a** Protein bar showing the canonical 422-amino acid human PAX6 protein, annotated with the ten commonest aniridia alleles. The domains and main secondary structural elements are shown (UnitProt P26367). The coding exons are 4–13, with exon boundaries indicted by a white vertical line. The location of exon 5a, part of the alternatively spliced isoform PAX6(5a), is indicated. **b** PAX6 paired domain bound to dsDNA. Note the two subdomains, each containing three alpha helices [PDB ID: 6PAX (Xu et al. 1999), from http://www.rcsb.org. Image of “6PAX” created using Protein Workshop (Moreland et al. 2005)]

The canonical human *PAX6* encodes a 422 amino acid, 46.7 kDa protein [UniProt P26367-1 (The UniProt Consortium 2017)], whilst the less abundant, alternatively spliced PAX6(5a) isoform (436 amino acid, 48.2 kDa, UniProt P26367-2) contains an extra 14 residues between Gln47 and Val48, encoded by exon 5a (Fig. 3a). PAX6 contains two DNA-binding domains, the bipartite paired domain (Fig. 3b) and the homeodomain, as well as a proline–serine–threonine-rich C-terminal transactivation domain. The paired and homeodomains can bind to DNA both cooperatively and independently, widening the repertoire of targets (Jun and Desplan 1996). A third isoform has been identified arising from an additional internal promoter (Carrière et al. 1995; Kim and Lauderdale 2006), though the role of such isoforms in humans, potential transcripts of which have been identified in silico, remains an interesting question (Bandah et al. 2008).

The subcellular localisation of PAX6 is predominantly nuclear. Nuclear localisation signals have been identified in the paired domain and near the homeodomain (Ploski et al. 2004; Tabata et al. 2018). However, non-nuclear localisation has also been observed. In chick retina, PAX6 was nuclear in the ganglion cell and inner nuclear layers, and cytoplasmic in the photoreceptor and outer nuclear layer (Shin et al. 2003), which may reflect its roles in diverse cellular processes (Simpson and Price 2002). In quail and nematode, paired-less isoforms are found in the cytoplasm (Carrière et al. 1995; Zhang et al. 1998). In the mouse brain, SPARC and Pax6 have been shown to co-localise and physically interact, and the authors suggest a model in which SPARC facilitates nuclear-cytoplasmic shuttling of Pax6 (Tripathi and Mishra 2010), a role that has also been shown for Karyopherin 13 (Ploski et al. 2004).

In PAX6(5a), the additional 14 amino acids between alpha helices 2 and 3 of the paired domain modulate its binding and transactivation potential, preferentially binding DNA with the C-terminal subdomain, acting as a molecular toggle (Epstein et al. 1994; Azuma et al. 1999). The ratio of PAX6(5a) to canonical PAX6 is higher in adult than embryonic tissue (Kozmik et al. 1997), with the PAX6(5a) isoform having a potential role in postnatal eye development (Singh et al. 2002). It is possible that other *PAX6* transcripts whose function is currently uncertain may also have a regulatory role.

PAX6 is thus able to increase its target diversity by alternative splicing as well as through the use of different DNA-binding domains (Simpson and Price 2002). Furthermore, fine-tuning is achieved through cooperative binding with other transcription factors, such as SOX2 (Kondoh et al. 2004; Hu et al. 2017). Targets of Pax6 include transcription factors [e.g., Six3 (Goudreau et al. 2002) and Foxc1 (Wang et al. 2016)], signalling molecules (e.g., Wnt) and crystallins [e.g., alpha A-crystallin (Cvekl et al. 1994)]; for targets in iris development see above. These *PAX6* target genes give rise to diverse ocular phenotypes when mutated in humans: missense mutations in *SIX3* cause microphthalmia, coloboma and holoprosencephaly (MIM #157170); *FOXC1* mutations cause anterior segment dysgenesis (MIM #601631), and alpha A-crystallin (*CRYAA)* mutations cause cataract (MIM #604219). Equally, heterozygous mutations of *SOX2, BMP4* and *MAB21L2* are associated with syndromic microphthalmia (MIM #206900, #607932 and #615877, respectively).

Some of the *PAX6* gene regulatory networks have been elucidated. An elegant and phenotypically relevant example is that reciprocal inhibition between Pax6 and Pax2 establishes the retinal-optic nerve boundary in mammals (Schwarz et al. 2000); *PAX2* mutation causes papillorenal syndrome in humans (MIM #120330). More recently, Pax6 was shown by ChIP to directly regulate *Prox1* and *Mab21l1* as part of a lens differentiation network (Sun et al. 2015).

Transcriptional activation of *PAX6* requires the coordinated activity of *cis*-regulatory elements, which are mostly conserved across mammals and zebrafish (Kleinjan et al. 2004; Lakowski et al. 2007). These were initially identified via human translocation cases, detailed below.

### *PAX6* mutation spectrum

Reflecting the many possible ways of disrupting PAX6 function, 472 unique sequence variants are reported (LOVD PAX6 database, version 170616, http://lsdb.hgu.mrc.ac.uk/home.php?select_db=PAX6). All classes of sequence variation are present and no domain of the protein is spared. The ten commonest aniridia variants are marked in Fig. 3. The wider range of phenotypes spans from mild iris hypoplasia to perinatal lethality.

#### Monoallelic mutations

##### Likely gene-disruptive variants

The commonest intragenic variants are encoded by single nucleotide substitutions resulting in a premature termination codon (PTC), with p.Arg240*, p.Arg317* and p.Arg203* being the three most frequently reported (129 reports in the LOVD database); these mutational hospots correspond to CpG dinucleotides (Tzoulaki et al. 2005). Frameshifting insertions, deletions and duplications, as well as many splice site mutations, also fall into this category of likely gene-disruptive variants. PTCs > 50–55 nucleotides upstream of the last coding exon–exon junction are predicted to be subject to nonsense-mediated decay (NMD). Though there could be exceptions to this NMD rule (Isken and Maquat 2007; Kervestin and Jacobson 2012), the known truncating mutations in humans phenocopy null alleles (Tzoulaki et al. 2005). In addition to the classical aniridia phenotype described above, some likely gene-disruptive variants appear to be hypomorphic, though milder phenotypes appear to be more common with missense variants (Hingorani et al. 2009).

##### Missense variants

Missense mutations in *PAX6*, which may lead to production of a full-length protein with altered structure and function (Mishra et al. 2002), have been published associated with both classical aniridia and non-classical phenotypes. These include optic nerve anomalies (Nallathambi et al. 2006), hypomorphic phenotypes such as *ectopia pupillae* (Hanson et al. 1999) and, more severely, microphthalmia (reviewed in Williamson and FitzPatrick 2014; Chassaing et al. 2014). In addition to canonical PAX6, two missense variants in the PAX6(5a) isoform have been described in five Japanese families (at least three unrelated) with congenital corneal opacification with or without foveal hypoplasia and cataract: p.5aGlu13Arg (1 case) (Nanjo et al. 2004), the rest p.5aVal7Asp, with functional work suggesting subtle changes in binding by PAX6(5a) and transactivation (Azuma et al. 1999). The structural and functional protein consequences of many of the missense variants in the database (in addition to many splice site and in-frame deletions) have not been determined experimentally. The functional consequences of missense mutations, unlike most PTCs, are dependent on their location within the protein. For example, missense mutations at the C-terminal amino acid of PAX6 impair transactivation mediated through the homeodomain (Singh et al. 2001).

##### C-terminal extensions

Frameshift duplications and substitutions predicted to result in C-terminal extensions are notably common (34 database reports; Fig. 3), some with variant phenotypes including exudative retinopathy (Hingorani et al. 2009).

##### Larger deletions and chromosomal rearrangements

Chromosomal rearrangements and whole/partial gene deletions disrupting *PAX6* account for approximately one-third of sporadic aniridia cases, and are also found in some familial cases (Crolla and van Heyningen 2002), such that overall these account for approximately 10% all aniridia cases (Robinson et al. 2008; Bobilev et al. 2015). Contiguous gene deletions causing WAGR syndrome are discussed below.

##### Variants disrupting the *cis*-regulation of PAX6

Via analysis of translocation and inversion breakpoints in aniridia patients, several disease-causing alleles were identified which disrupt the long-range regulation of *PAX6*. These were located 11 kb, 22 kb (Lauderdale et al. 2000), 125 kb and 150 kb (SIMO element) (Simola et al. 1983; Fantes et al. 1995) downstream (telomeric) to *PAX6*. Analysis of the SIMO element showed that this is an autoregulatory binding site whose disruption impairs PAX6 maintenance (Bhatia et al. 2013). The phenotypes were identical to heterozygous deletion or PTC-causing mutations of *PAX6*. Mutations in *PAX6* regulatory regions are now more easily detectable by array-based comparative genomic hybridisation. Ansari et al. identified five aniridia patients with deletions involving plausible regulatory regions and suggest a ‘critical region’ for transcriptional activation spanning 244 kb (Ansari et al. 2016).

#### Biallelic mutations

Biallelic mutations in *PAX6* with two likely gene-disruptive alleles cause severe CNS defects with anophthalmia and perinatal lethality (Glaser et al. 1994; Schmidt-Sidor et al. 2009). A similarly severe ocular phenotype was reported in a viable compound heterozygous child with trisomy 21 (Solomon et al. 2009). Two viable compound heterozygotes families have been published with aniridia or coloboma, and no severe systemic effects, through having a combination of a likely gene-disruptive and presumed mildly hypomorphic missense allele (Grønskov et al. 1999; Chao et al. 2003).

### Other forms of *PAX*6-related eye disease

Distinct phenotypes described in association with *PAX6* mutations include Peters anomaly and other anterior segment dysgeneses (Prokudin et al. 2014), coloboma and microphthalmia (Williamson and FitzPatrick 2014), optic nerve anomalies (Azuma et al. 2003; Nallathambi et al. 2006), *ectopia pupillae* and nystagmus (Hanson et al. 1999). Commonly seen are the non-iris features of classical aniridia, either in isolation or combination, namely cataract, foveal hypoplasia, glaucoma and keratopathy (Sale et al. 2002; Hever et al. 2006). Most of these phenotypes can be considered variable expressivity of classical aniridia.

### Genetic differential diagnosis of classical aniridia

Whilst classical aniridia is still largely considered a monogenic disorder, rarely aniridia-like phenotypes have occurred with mutations in two other anterior segment disease genes. These include two cases with *PITX2* mutations—one intragenic (Perveen et al. 2000) and one with a telomeric regulatory mutation (Ansari et al. 2016)—and seven cases with *FOXC1* mutations. Three of the latter are missense cases (Ito et al. 2009; Ansari et al. 2016), and four are whole gene deletions (Sadagopan et al. 2015; Ansari et al. 2016). Of these nine aniridia cases, seven had known congenital glaucoma (one unknown), many presenting as infants with buphthalmos, suggesting that this is more common in *FOXC1-* than *PAX6-*associated aniridia.

An aniridia family with a segregating missense mutation in *TRIM44*, ~ 4 Mb away from *PAX6* on 11p13, was described (Zhang et al. 2015), but we advise caution in ascribing pathogenicity as this is a single pedigree supported by in vitro functional data. There remains a small but significant proportion of aniridia patients with no genetic diagnosis (Ansari et al. 2016).

Following an analysis of 42 *PAX6*-negative aniridia cases with array CGH (comparative genomic hybridisation), which identified some of the cases above (Ansari et al. 2016), the authors were left with 27/42 unexplained cases. Since this publication, the Gillespie syndrome cases in this cohort have been shown to have *ITPR1* mutations (McEntagart et al. 2016; Gerber et al. 2016). We estimate the proportion of unexplained classical aniridia cases to be approximately 5%.

## Contiguous deletion syndromes encompassing *PAX6*: WAGR syndrome

### Background and clinical features

In 1964, Robert Miller et al. reported the results of a review of the case records of 440 individuals with Wilms tumour, a rare malignancy of embryonic origin affecting the kidney (Miller et al. 1964). Six of these children (1.4%) also had aniridia, which was unlikely to be a coincidence given the incidence of aniridia at that time of 1 in 50,000 live births (Shaw et al. 1960). Each of the children with Wilms tumour and aniridia also had significant intellectual disability or global developmental delay (termed mental retardation at the time). Intellectual disability is not a common association in isolated Wilms tumour or classical aniridia. Three of the six children had microcephaly and one child had contralateral renal hypoplasia. Subsequent to this original report, many other cases were published. This constellation of clinical features has been named either WAGR syndrome (Wilms tumour, aniridia, genitourinary anomalies, mental retardation) or AGR syndrome, depending on the presence or absence of Wilms tumour. For simplicity, we will use WAGR syndrome to cover both groups.

Remarkably few case series or systematic reviews have been published which focus on the clinical aspects of WAGR. The most useful is a series of 54 clinically well-characterised individuals (31 males, 23 females) with WAGR (Fischbach et al. 2005). Clinical features observed in more than two individuals in this series are listed in Table 2. Of these non-canonical WAGR features, obesity has generated the most clinical interest (Marlin et al. 1994; Amor 2002) and has led to the suggestion that WAGR be re-designated as WAGRO (Tiberio et al. 2000).

**Table 2:** Clinical features associated with WAGR+/−O (excluding aniridia, Wilms tumour and intellectual disability): non-canonical WAGR features observed in > 2 individuals in a series of 54 WAGR cases (Fischbach et al. 2005)

System	Feature	%
Genitourinary	Cryptorchidism	61.3
Respiratory	Recurrent sinusitis	27.8
Renal	Proteinuria	25.9
Behavioural	Short attention span	22.2
Respiratory	Obstructive sleep apnoea	20.4
Behavioural	Autism spectrum disorders	18.5
Metabolic	Obesity	18.5
Dental	Severe dental malocclusion	16.7
Ocular	Optic nerve hypoplasia	14.8
Musculoskeletal	Scoliosis/kyphosis	14.8
Neurologic	Hypertonia/hypotonia	13
Genitourinary	Hypospadias	12.9
Renal	Focal segmental glomerulosclerosis	11.1
Respiratory	Recurrent pneumonia	11.1
Ocular	Retinal detachment	9.3
Ocular	Strabismus	7.4
Genitourinary	Ambiguous genitalia	7.4
Neurologic	Epilepsy	7.4
Genitourinary	Inguinal hernia	5.6
Musculoskeletal	Hemihypertrophy	5.6
Metabolic	Hyperlipidemia	5.6
Abdominal	Chronic pancreatitis	5.6

### Cytogenetic analysis

**Fig. 4:**
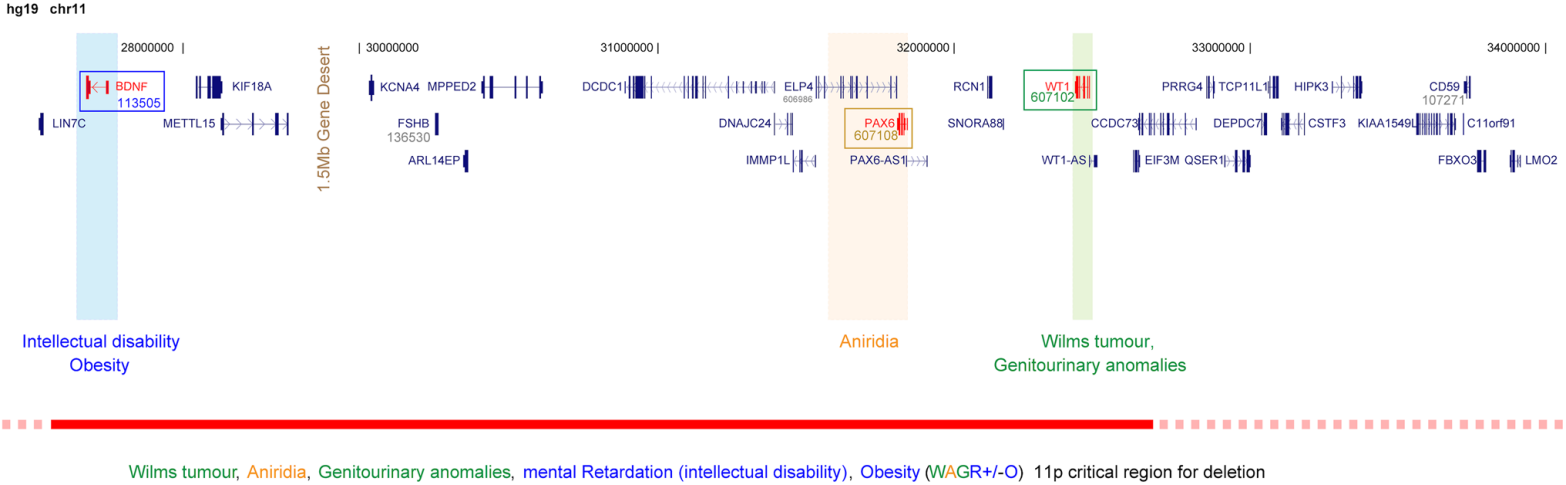
Molecular pathology of WAGR syndrome. Cartoon of the 11p genomic region which encompasses *PAX6, WT1* and *BDNF*. The numbers and lines at the top represent the hg19 human genomic coordinates, below which are the gene models. If the gene is an OMIM morbid gene the cognate OMIM number is given below the gene symbol. The boxed genes are those that have been confidently associated with components of the WAGR syndrome. The bar at the bottom is an empirically-derived critical region for deletions associated with WAGR. However, it is likely there are many possible causes of the intellectual disability associated with WAGR, so it is reasonable to consider that any deletion which encompasses *PAX6* and *WT1* may be WAGR-associated

Chromosome 11p13 deletions were first identified in three individuals with WAGR in 1978 (Riccardi et al. 1978). Following detailed mapping of this chromosome region, the causative genes for Wilms tumour and genitourinary anomalies (*WT1*) (Gessler et al. 1990) and classical aniridia (*PAX6*) (Ton et al. 1991) were found to lie within a megabase of each other (Fig. 4). The term “contiguous deletion syndrome” is used to describe distinctive patterns of clinical features caused by haploinsufficiency for more than one gene mapping to the same genomic locus encompassed by a chromosome deletion. WAGR is the paradigm for this type of genetic disorder.

*WT1* was identified via homozygous intragenic deletions in isolated Wilms tumours cases (Gessler et al. 1990). This was consistent with *WT1* being a tumour suppressor gene operating in a manner consistent with the “two hits hypothesis” (Knudson 1971; Haber et al. 1990), which requires inactivation of both copies of the gene in the relevant tissue to initiate tumourigenesis. *WT1* encodes a zinc finger protein (Wilms tumour protein, UniProt P19544) which functions as a transcription factor and an RNA-binding protein. Heterozygous intragenic, dominant-negative mutations in *WT1* have been identified in several overlapping developmental disorders characterised by Wilms tumour, genital malformations, male-to-female sex reversal, nephropathy, diaphragmatic hernia and gonadoblastoma (Pelletier et al. 1991; Hastie 1992).

The gene—or genes—responsible for the cause of the neurodevelopmental problems and obesity in WAGR is not yet confirmed. Several lines of evidence implicate the gene encoding brain-derived neurotrophic factor (BDNF) in both these components of WAGR (Xu et al. 2008; Han et al. 2008, 2013; Shinawi et al. 2011; Rodríguez-López et al. 2013). Heterozygous *Bdnf* knockout mice have hyperphagia, obesity and poorer learning and social behaviours (Lyons et al. 1999; Kernie et al. 2000). In humans, WAGR patients with *BDNF* haploinsufficiency had reduced cognitive functioning, lower adaptive behaviour and higher levels of obesity, compared to those without a *BDNF* deletion (Han et al. 2008, 2013). The *BDNF* locus was also identified as a GWAS hit for obesity (Wen et al. 2012). The genes *SLC1A2* and *PRRG4* have also been implicated in the neurodevelopmental component (Xu et al. 2008). *PAX6* itself has also been proposed, given its known involvement in CNS development and the small proportion of cases with intragenic *PAX6* mutations associated with developmental delay or autism (collated by Davis et al. 2008; Chien et al. 2009).

### Relevance to isolated aniridia

Aniridia is usually apparent from soon after birth. Although WAGR-associated deletions represent a rare subset of causative alleles resulting in haploinsufficiency for *PAX6*, it is important to identify such deletions as soon as possible as they have a significant impact on both prognosis and management during infancy and beyond (Fig. 6). Most obviously, individuals who have deletions encompassing both *PAX6* and *WT1* should be screened for Wilms tumour as earlier detection almost certainly improves outcome (Pritchard-Jones et al. 2016). Due to the high incidence of end-stage renal failure in this group (Fischbach et al. 2005), lifelong monitoring of renal function is recommended. It is also reasonable for an individual with a WAGR deletion to be followed up by a paediatrician with expertise in the early recognition and management of intellectual disability and autism.

## Gillespie syndrome

### Clinical features

Apparent partial aniridia, presenting as congenital mydriasis, is an invariant feature of Gillespie syndrome (GS; OMIM 206700), in association with non-progressive cerebellar hypoplasia and ataxia, intellectual disability and congenital hypotonia. In GS the iris defect is bilateral and has a distinct scalloped appearance resulting from aplasia of the tissue central to the collarette (Fig. 2b). This aplasia appears to be a consequence of abnormal development of the ectoderm-derived sphincter musculature and the surrounding mesoderm-derived stromal tissues at the distal tip of the foetal iris. In contrast to *PAX6*-associated aniridia, the ocular defects in GS are essentially restricted to the iris and the variable presence of iridolenticular strands. Foveal hypoplasia is rarely observed (McEntagart et al. 2016).

Additional phenotypes are noted infrequently, but multiple cases have shown cardiac (pulmonary valve stenosis, patent foramen ovale), skeletal (affecting the vertebrae or digits), and gastro-oesophageal defects (Dentici et al. 2017; reviewed by Carvalho et al. 2017). The early clinical reports of this syndrome were limited to 12 families (reviewed by Mariën et al. 2008), and they supported X-linked, autosomal recessive and autosomal dominant modes of inheritance.

### Identification of *ITPR1* as the causal gene

In 2016, two independent projects utilising whole exome sequencing concurrently identified *ITPR1* (inositol 1,4,5-trisphosphate receptor type 1; OMIM 147265; hg38 chr3:4493348–4847840) as the causative gene in GS (McEntagart et al. 2016; Gerber et al. 2016). These projects studied one familial mother–daughter transmission (Mariën et al. 2008) and 15 singleton cases, with a further five cases reported subsequently (Zambonin et al. 2017; Dentici et al. 2017; Carvalho et al. 2017). GS is now considered a monogenic disorder, as all 22 reported cases with genetic testing had monoallelic or biallelic pathogenic mutations identified in *ITPR1*. Interestingly, 17 of these cases were known to be female, although such a marked sex bias was not evident in the families described in the early clinical reports with no genetic diagnosis. There is no evidence for non-penetrance for *ITPR1* mutations in GS: all individuals with a pathogenic mutation are affected.

### ITPR1 biology and structure

The ITPR1 receptor is a type I intracellular transmembrane calcium release channel that is inositol 1,4,5-trisphosphate (IP3) responsive [UniProt Q14643, alternative names IP3R1, InsP3R1; reviewed by Patel et al. (1999)]. The subcellular localisation of the receptor is perinuclear: it is primarily in the smooth endoplasmic reticulum (ER), though intranuclear, possibly nucleoplasm reticular, ITPR1 foci have also been reported (Maeda et al. 1990; McEntagart et al. 2016). The ITPR1 protein complex is a homotetramer: each subunit is oriented with a large N-terminal region, containing an IP3-ligand binding site, and a short C-terminal region, both in the cytoplasm; six transmembrane domains contributing to the channel pore in the ER membrane, and inter-transmembrane domain loops in the ER lumen (Jiang et al. 2002). The different subtypes of ITPR are ubiquitously expressed, with high levels of ITPR1 in the brain and particularly in the Purkinje cells of the cerebellum (Maeda et al. 1990). The expression pattern in the developing iris is not reported. ITPR1 channel function shows calcium-dependent increased inhibition when the receptor is bound to the ITPR interacting protein (ITPRIP, previously called DANGER; UniProt Q8IWB1), which is highly co-expressed in Purkinje cells and contains an ER/plasma membrane signal peptide and a conserved partial Mab21 domain (van Rossum et al. 2006). In mouse models of *Itpr1* null alleles, heterozygosity elicits mildly impaired motor coordination and minor iris defects distinct from those of GS (Ogura et al. 2001; McEntagart et al. 2016), and homozygosity is embryonic lethal or causes severe ataxia and seizures with early death (Matsumoto et al. 1996; reviewed by Tada et al. 2016).

### *ITPR1* mutation spectrum

**Fig. 5:**
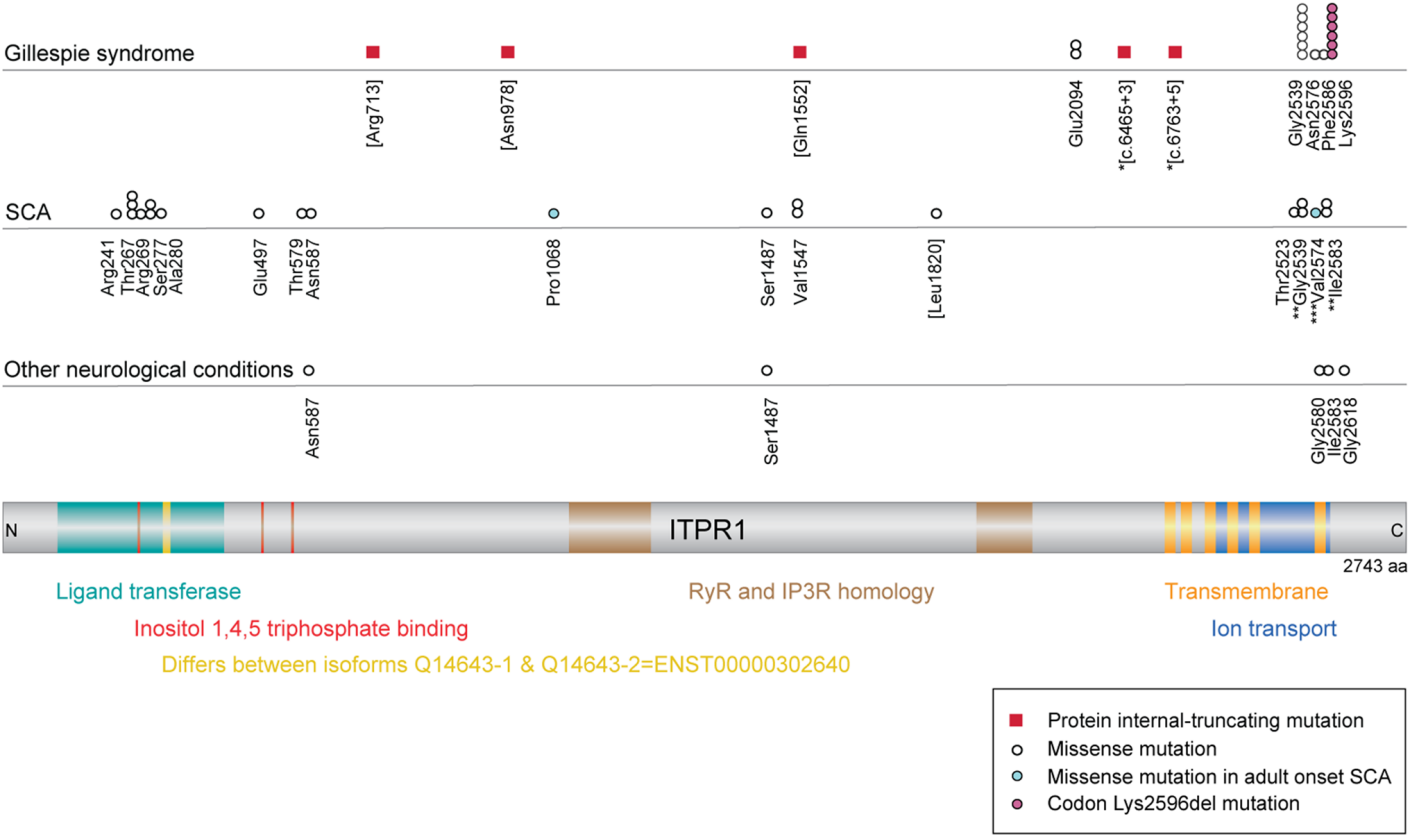
Spectrum of ITPR1 pathogenic variants. A linear protein schematic of ITPR1 is shown in the bottom panel, with domains and features demarcated and labelled in colour. The position of the 15-amino acid insertion in the longer isoform, UniProt Q14643-1, is shown in yellow. Amino acid numbering and domain positions are based on the 2743-amino acid isoform 2: Q14643-2, encoded by the canonical transcript GenBank NM_001168272.1; ENST00000302640. Shown above the protein schematic are all of the published variants associated with Gillespie syndrome and other neurological conditions, and all of the published substitution variants associated with spinocerebellar ataxia (SCA). All variants shown have had their amino acid numbering unified to isoform 2 (to facilitate an accurate collation and comparison of the ITPR1 dataset) and may therefore differ from the numbering used in the original publication. Variants in brackets indicate recessive alleles, of which *compound heterozygous alleles identified in a single proband; **cases reported as having a SCA29 phenotype and with substitutions at residues associated with Gillespie syndrome (identical variant) or pontocerebellar hypoplasia (different variant); ***variant associated with adult onset SCA in a mother and early onset SCA in her daughter

In GS, *ITPR1* mutations have been identified in four families with recessive homozygous or compound heterozygous alleles, and in 16 families with dominant heterozygous alleles (McEntagart et al. 2016; Gerber et al. 2016; Zambonin et al. 2017; Dentici et al. 2017; Carvalho et al. 2017). The families with dominant mutations include the one familial case, with mother–daughter transmission, and a de novo event in a consanguineous family. Parental DNA was available for all but one of the remaining dominant families, and de novo occurrence of the mutation was established in each case. The recessive alleles are predicted to generate premature termination codons that, possibly via exon skipping mechanisms, appear to act as partial rather than full loss-of-function mutations; as the carrier parents of these alleles are unaffected there is no apparent haploinsufficient effect associated with these hypomorphic variants (see below for *ITPR1* haploinsufficiency in non-GS ataxia; Fig. 5).

Given that less than 50% of normal levels of ITPR1 are necessary to cause GS, it was proposed that the dominant heterozygous alleles associated with this syndrome are not loss-of-function mutations but are dominant-negative mutations. These dominant alleles comprise missense mutations or codon-Lys2596 deletion, and alter C-terminal residues that are located at or near the ER transmembrane domain and the associated ion-transport region of the channel. The restricted and recurrent nature of the dominant Lys2596 deletion and missense mutations, which substitute residues Glu2094, Gly2539, Asn2576, or Phe2586 in the pore of the channel (Fig. 5), indicates that these residues are critical for ITPR1 function (all numbering from RefSeq Q14643-2, isoform 2; 2743 amino acids). In contrast to other pathogenic variants, these dominant-negative variants are thought to be less destabilising and thereby permit ITPR1 protein complex formation but with abrogated function (McEntagart et al. 2016; Natan et al. 2016). Furthermore, as a consequence of the homotetrameric structure of ITPR1, these mutations are predicted to generate variant subunits that will incorporate in to 15/16 protein complexes formed. Decreased calcium release activity was detected in *ITPR1*(Lys2596deletion)-mutant transfected cells; however, the in vivo mechanism(s) underlying the altered function of the mutant protein complex in recessive and dominant GS is not known (Gerber et al. 2016).

### Other forms of *ITPR1-*associated disease

Pathogenic mutations in *ITPR1* were first described in two types of spinocerebellar ataxia (SCA), in which iris development is normal. Both of these SCA types are essentially dominant conditions. The late onset, slowly progressive form, SCA15 (OMIM 606658), results from *ITPR1* haploinsufficiency caused mainly by whole gene deletion, but also from stop-gain or rare missense mutations (reviewed by Tada et al. 2016). The congenital/infantile non-progressive form, SCA29 (OMIM 117360), is caused by heterozygous in-frame, mostly missense, mutations that alter residues predominantly in the cytoplasmic N-terminal portion of ITPR1 (reviewed by Zambonin et al. 2017). A recessive variant, Leu1820Pro, was recently identified in a SCA29 consanguineous family, where the carrier individuals had asymptomatic cerebellar hypoplasia (Klar et al. 2017). Furthermore, “GS-like” heterozygous variants that substitute residues in the ion-transport channel have been identified in cases with SCA (including reported SCA29 cases with the recurrent GS variant Gly2539Arg) (Valencia et al. 2015; Zambonin et al. 2017; Hsiao et al. 2017), pontocerebellar hypoplasia (Hayashi et al. 2017; van Dijk et al. 2017) or peripheral neuropathy (Gonzaga-Jauregui et al. 2015). De novo substitution variants have also been identified in ataxic cerebral palsy cases with normal neuroimaging (Parolin Schnekenberg et al. 2015) (Fig. 5). These reports demonstrate that recessive and dominant alleles of *ITPR1* are now associated with both GS and SCA, and that additional specific alleles can cause other neurological conditions. The genotype:phenotype pattern is diverse and indicates that the ITPR1 protein complex has multiple functional regions that are intricately tissue-specific and that can be aberrantly altered by numerous mechanisms.

### Differential diagnosis of Gillespie syndrome

Prior to the identification of *ITPR1* mutations causing GS, two aniridia cases with GS features were reported with *PAX6* mutations: one had a non-coding exon essential-splice mutation and no ataxia or cerebellar abnormalities (Ticho et al. 2006), and the second, with a typical *PAX6* stop-gain mutation, had ataxia but no cerebellar abnormalities (Graziano et al. 2007). In both cases, the iris remnant did not have the scalloped appearance that is characteristic of GS.

A GS-like iris defect is evident in the fixed dilated pupils of individuals with a severe multisystemic smooth muscle dysfunction syndrome (OMIM 613834) caused by Arg179 substitutions in the *ACTA2* gene (actin, alpha 2, smooth muscle, aorta; OMIM 102620; Fig. 2c) (Roulez et al. 2014; reviewed by; Regalado et al. 2018). Cyclic GMP kinase signalling complex studies in tracheal smooth muscle have indicated an interaction between ITPR1 and alpha-actin (Koller et al. 2003); however, functional analyses of these two proteins in iris tissue are not reported.

## Investigations and clinical management

**Fig. 6:**
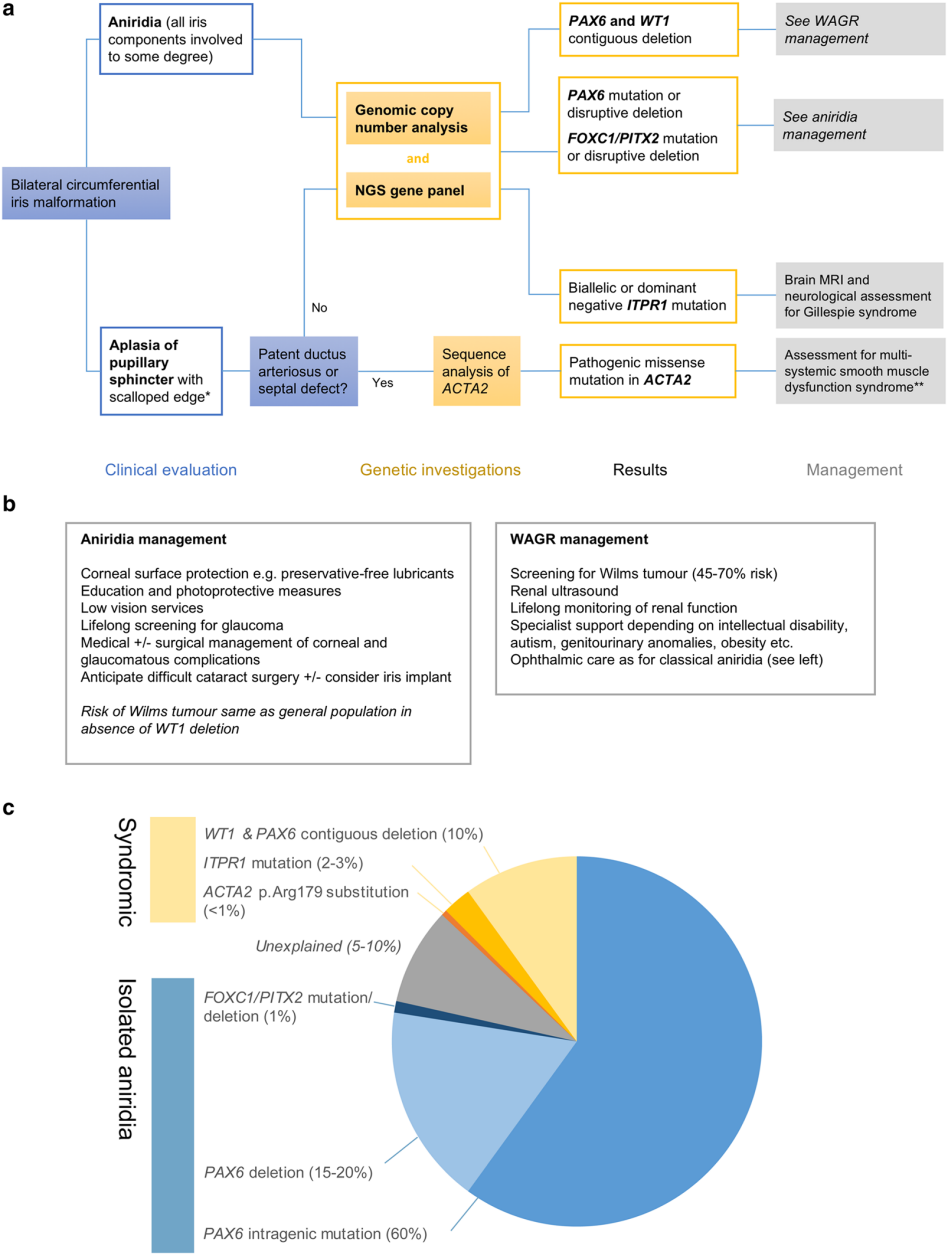
**a** Flowchart showing an example approach to the genetic investigation of new congenital aniridia/congenital mydriasis cases. This will usually consist of an aniridia NGS gene panel—in the UK this includes *PAX6, FOXC1, PITX2* and *ITPR1*—and copy number analysis (e.g., chromosomal array or array-based comparative genomic hybridisation). The latter is important to look for contiguous deletion of *PAX6* and *WT1*. We have suggested the combination of iris and cardiac features which should prompt consideration of *ACTA2* sequencing—this could be tested with other genes such as *ITPR1* depending on clinical judgement. *Gillespie syndrome-like iris (see Fig. 2b, c); **a suggested surveillance regimen for the serious complications of *ACTA2* multisystemic smooth muscle dysfunction has recently been outlined (Regalado et al. 2018). **b** Brief outline of the management of aniridia and WAGR syndrome. **c** Pie chart showing the genetic causes of isolated and syndromic aniridia. Note that these frequencies are calculated as a percentage of all aniridia cases (using the term broadly and including Gillespie syndrome). For example, whilst *ITPR1* accounts for 2–3% aniridia cases, it is the only known cause of Gillespie syndrome. They are estimated and approximate frequencies derived from published sources (Grønskov et al. 2001; Crolla and van Heyningen 2002; Robinson et al. 2008; Bobilev et al. 2015; Ansari et al. 2016) or, where no published data is available, estimated from our cohort of > 400 aniridia patients

The investigation and management of aniridia cases are multidisciplinary, usually involving genetics, paediatric and ophthalmology services, and even more so in syndromic cases. An example approach to working up new aniridia/congenital mydriasis cases is shown in Fig. 6, with general management recommendations. For the vast majority of aniridia cases, this comprises two tests: an NGS gene panel (that ideally includes *PAX6, FOXC1, PITX2* and *ITPR1*) and an assessment of copy number such as array CGH. The array is important to detect deletions encompassing *WT1*, which determine the need for continuing Wilms tumour screening (Grønskov et al. 2001) and monitoring of renal function. In familial aniridia cases, the chance of a *WT1* deletion is very low, with only two WAGR syndrome familial cases reported (Fantes et al. 1992; Robinson et al. 2008). However, the array retains excellent diagnostic value, as the high-resolution assessment of copy number can detect whole gene or regulatory region deletions in *PAX6* (or rarely, *FOXC1*/*PITX2)* (Ansari et al. 2016; Blanco-Kelly et al. 2017; Franzoni et al. 2017). We therefore have not distinguished between the investigation of sporadic and familial aniridia cases, though clearly in familial cases where the genetic cause is already known, this should be tested directly.

There are two causes of aniridia associated with life-limiting complications. In WAGR syndrome, Wilms tumour survival rates approach 95% at 4 years, but WAGR carries a late mortality risk from end-stage renal failure (Breslow et al. 2003; Brok et al. 2017). The International WAGR Syndrome Association produce a comprehensive management summary for physicians including screening protocols (http://wagr.org). Individuals with *ACTA2* multisystemic smooth muscle dysfunction are at risk of acute, life-threatening vascular events including aortic dissections and ischaemic strokes in childhood (Roulez et al. 2014); it is important to identify these rare cases early to instigate appropriate surveillance (Regalado et al. 2018).

For patients with a Gillespie syndrome-like iris (Fig. 2b, c), the two known genetic causes are *ITPR1* and *ACTA2* p.Arg179 substitutions. In most cases, the iris defect is detected before the other features of these conditions. However, there is one clinical feature which can aid the decision of whether to test *ACTA2* in addition to *ITPR1*. In the largest series of *ACTA2* p.Arg179 cases (33 individuals), the only feature reliably present at birth in all cases, other than the iris defect, was cardiac: a large patent ductus arteriosus (PDA, 91%) or aortopulmonary septal defect (9%). Based on this and given the serious prognostic implications, it seems reasonable to only test *ACTA2* if both the iris and cardiac features are present.

One of the principal benefits of NGS gene panels for patients with unexplained ocular disease is the number of eye genes that can be tested simultaneously and cost effectively. For example, known *PAX6* target genes, such as *BMP4, FOXC1* and *SIX3* discussed above—which are not aniridia genes but cause diverse eye malformations—can be included in such a panel.

If all of the suggested investigations are negative, options for pursuing further investigation include looking for balanced chromosome translocations and whole exome or whole genome sequencing; depending on availability, the latter may be more often pursued in a research rather than diagnostic laboratory setting. In addition to the management outlined in Fig. 6, optical coherence tomography (OCT) is valuable for evaluating foveal hypoplasia, though this is postponed until the child can cooperate. Furthermore, MRI brain may be helpful in classical aniridia if there are relevant neurological symptoms (e.g., olfactory, auditory), as well as in Gillespie syndrome. Lastly, it is also helpful to alert families to the existence of patient organisations, such as Aniridia Network (http://aniridia.org.uk), Aniridia Europe (http://www.aniridia.eu), Aniridia Foundation International (http://www.aniridia.net) and IWSA (http://wagr.org).

Knowledge of the specific mutation in aniridia may become important for determining eligibility for future therapies. Ataluren, which targets in-frame nonsense mutations and showed promise in a *Sey* mouse model with a nonsense mutation in the linker region (Gregory-Evans et al. 2014), is in phase 2 clinical trials (STAR trial, ClinicalTrials.gov ID NCT02647359) (Wang and Gregory-Evans 2015). Ataluren failed to meet the primary endpoint in phase 3 trials for cystic fibrosis (Kerem et al. 2014) and Duchenne muscular dystrophy (McDonald et al. 2017), but showed benefit in a subgroup of the latter. New treatments for the complications of aniridia are also undergoing evaluation. Surgical treatments at the clinical trial stage for aniridia include oral mucosal epithelial transplantation (EU Clinical Trials Register ID 2011-000598-30) and ex vivo expanded corneal limbal stem cell transplantation (UK Clinical Trial Gateway ID, ISRCTN54055321). Both of these aim to treat aniridic keratopathy, the principal cause of progressive visual loss, and as such have the potential to improve quality of life of individuals with aniridia.

## Conclusion

In summary, whilst the story of classical aniridia genetics took form nearly three decades ago with the identification of *PAX6*, the cause for Gillespie syndrome has only recently emerged as *ITPR1*. The role of the latter in iris development is not yet clear, and equally many *PAX6-*dependent gene regulatory networks remain poorly understood. Just as *PAX6* has served as a model for dosage-sensitive transcription factors, WAGR syndrome is a paradigm for contiguous deletion syndromes. We have outlined a genetic investigation strategy for aniridia and Gillespie syndrome, including copy number analysis and gene panel testing, and suggest criteria for testing *ACTA2*. Lastly, an estimated 5% of classical aniridia remains unexplained. These cases may be caused by novel mechanisms that disrupt the known genes or by mutations at new loci, which may be elucidated by whole genome sequencing studies.

## References

[CR1] Aalfs CM, Fantes JA, Wenniger-Prick LJJM (1997). Tandem duplication of 11p12–p13 in a child with borderline development delay and eye abnormalities: dose effect of the PAX6 gene product?. Am J Med Genet.

[CR2] Adams D, Karolak M, Robertson E, Oxburgh L (2007). Control of kidney, eye and limb expression of Bmp7 by an enhancer element highly conserved between species. Dev Biol.

[CR3] Amor DJ (2002). Morbid obesity and hyperphagia in the WAGR syndrome. Clin Dysmorphol.

[CR4] Ansari M, Rainger J, Hanson IM (2016). Genetic analysis of “PAX6-Negative” individuals with aniridia or gillespie syndrome. PLoS One.

[CR5] Azuma N, Yamaguchi Y, Handa H (1999). Missense mutation in the alternative splice region of the PAX6 gene in eye anomalies. Am J Hum Genet.

[CR6] Azuma N, Yamaguchi Y, Handa H (2003). Mutations of the PAX6 gene detected in patients with a variety of optic-nerve malformations. Am J Hum Genet.

[CR7] Bamiou D-E, Free SL, Sisodiya SM (2007). Auditory interhemispheric transfer deficits, hearing difficulties, and brain magnetic resonance imaging abnormalities in children with congenital aniridia due to PAX6 mutations. Arch Pediatr Adolesc Med.

[CR8] Bandah D, Rosenmann A, Blumenfeld A (2008). A novel de novo PAX6 mutation in an Ashkenazi-Jewish family with aniridia. Mol Vis.

[CR9] Bao ZZ, Cepko CL (1997). The expression and function of Notch pathway genes in the developing rat eye. J Neurosci.

[CR10] Barratta G (1818) Osservazioni pratiche sulle principali malattie degli occhi. Milan

[CR11] Bhatia S, Bengani H, Fish M (2013). Disruption of autoregulatory feedback by a mutation in a remote, ultraconserved PAX6 enhancer causes aniridia. Am J Hum Genet.

[CR12] Blanco-Kelly F, Palomares M, Vallespín E (2017). Improving molecular diagnosis of aniridia and WAGR syndrome using customized targeted array-based CGH. PLoS One.

[CR13] Bobilev AM, McDougal ME, Taylor WL (2015). Assessment of PAX6 alleles in 66 families with aniridia. Clin Genet.

[CR14] Breslow NE, Norris R, Norkool PA (2003). Characteristics and outcomes of children with the Wilms tumor-Aniridia syndrome: a report from the National Wilms Tumor Study Group. J Clin Oncol.

[CR15] Brok J, Pritchard-Jones K, Geller JI, Spreafico F (2017). Review of phase I and II trials for Wilms’ tumour—can we optimise the search for novel agents?. Eur J Cancer.

[CR16] Carrière C, Plaza S, Caboche J (1995). Nuclear localization signals, DNA binding, and transactivation properties of quail Pax-6 (Pax-QNR) isoforms. Cell Growth Differ.

[CR17] Carvalho DR, Medeiros JEG, Ribeiro DSM (2017). Additional features of Gillespie syndrome in two brazilian siblings with a novel ITPR1 homozygous pathogenic variant. Eur J Med Genet.

[CR18] Chang B, Smith RS, Peters M (2001). Haploinsufficient Bmp4 ocular phenotypes include anterior segment dysgenesis with elevated intraocular pressure. BMC Genet.

[CR19] Chao L-Y, Mishra R, Strong LC, Saunders GF (2003). Missense mutations in the DNA-binding region and termination codon in PAX6. Hum Mutat.

[CR20] Chassaing N, Causse A, Vigouroux A (2014). Molecular findings and clinical data in a cohort of 150 patients with anophthalmia/microphthalmia. Clin Genet.

[CR21] Chien Y-H, Huang H-P, Hwu W-L (2009). Eye anomalies and neurological manifestations in patients with PAX6 mutations. Mol Vis.

[CR22] Crolla JA, van Heyningen V (2002). Frequent chromosome aberrations revealed by molecular cytogenetic studies in patients with aniridia. Am J Hum Genet.

[CR23] Cvekl A, Callaerts P (2017). PAX6: 25th anniversary and more to learn. Exp Eye Res.

[CR24] Cvekl A, Tamm ER (2004). Anterior eye development and ocular mesenchyme: new insights from mouse models and human diseases. Bioessays.

[CR25] Cvekl A, Sax CM, Bresnick EH, Piatigorsky J (1994). A complex array of positive and negative elements regulates the chicken alpha A-crystallin gene: involvement of Pax-6, USF, CREB and/or CREM, and AP-1 proteins. Mol Cell Biol.

[CR26] Davis LK, Meyer KJ, Rudd DS (2008). Pax6 3′ deletion results in aniridia, autism and mental retardation. Hum Genet.

[CR27] Davis-Silberman N, Ashery-Padan R (2008). Iris development in vertebrates; genetic and molecular considerations. Brain Res.

[CR28] Dentici ML, Barresi S, Nardella M (2017). Identification of novel and hotspot mutations in the channel domain of ITPR1 in two patients with Gillespie syndrome. Gene.

[CR29] Douvaras P, Mort RL, Edwards D (2013). Increased corneal epithelial turnover contributes to abnormal homeostasis in the Pax6(+/) mouse model of aniridia. PLoS One.

[CR30] Eden U, Iggman D, Riise R, Tornqvist K (2008). Epidemiology of aniridia in Sweden and Norway. Acta Ophthalmol.

[CR31] Epstein JA, Glaser T, Cai J (1994). Two independent and interactive DNA-binding subdomains of the Pax6 paired domain are regulated by alternative splicing. Genes Dev.

[CR32] Fantes JA, Bickmore WA, Fletcher JM (1992). Submicroscopic deletions at the WAGR locus, revealed by nonradioactive in situ hybridization. Am J Hum Genet.

[CR33] Fantes J, Redeker B, Breen M (1995). Aniridia-associated cytogenetic rearrangements suggest that a position effect may cause the mutant phenotype. Hum Mol Genet.

[CR34] Fischbach BV, Trout KL, Lewis J (2005). WAGR syndrome: a clinical review of 54 cases. Pediatrics.

[CR35] Franzoni A, Russo PD, Baldan F (2017). A CGH array procedure to detect PAX6 gene structural defects. Mol Cell Probes.

[CR36] Free SL, Mitchell TN, Williamson KA (2003). Quantitative MR image analysis in subjects with defects in the PAX6 gene. Neuroimage.

[CR37] Gerber S, Alzayady KJ, Burglen L (2016). Recessive and dominant de novo ITPR1 mutations cause gillespie syndrome. Am J Hum Genet.

[CR38] Gessler M, Poustka A, Cavenee W (1990). Homozygous deletion in Wilms tumours of a zinc-finger gene identified by chromosome jumping. Nature.

[CR39] Glaser T, Lane J, Housman D (1990). A mouse model of the aniridia-Wilms tumor deletion syndrome. Science.

[CR40] Glaser T, Jepeal L, Edwards JG (1994). PAX6 gene dosage effect in a family with congenital cataracts, aniridia, anophthalmia and central nervous system defects. Nat Genet.

[CR41] Gonzaga-Jauregui C, Harel T, Gambin T (2015). Exome sequence analysis suggests that genetic burden contributes to phenotypic variability and complex neuropathy. Cell Rep.

[CR42] Goudreau G, Petrou P, Reneker LW (2002). Mutually regulated expression of Pax6 and Six3 and its implications for the Pax6 haploinsufficient lens phenotype. Proc Natl Acad Sci USA.

[CR43] Grant WM, Walton DS (1974). Progressive changes in the angle in congenital aniridia, with development of glaucoma. Am J Ophthalmol.

[CR44] Graziano C, D’Elia AV, Mazzanti L (2007). A de novo nonsense mutation of PAX6 gene in a patient with aniridia, ataxia, and mental retardation. Am J Med Genet A.

[CR45] Gregory-Evans CY, Wang X, Wasan KM (2014). Postnatal manipulation of Pax6 dosage reverses congenital tissue malformation defects. J Clin Invest.

[CR46] Grønskov K, Rosenberg T, Sand A, Brøndum-Nielsen K (1999). Mutational analysis of PAX6: 16 novel mutations including 5 missense mutations with a mild aniridia phenotype. Eur J Hum Genet.

[CR47] Grønskov K, Olsen JH, Sand A (2001). Population-based risk estimates of Wilms tumor in sporadic aniridia. A comprehensive mutation screening procedure of PAX6 identifies 80% of mutations in aniridia. Hum Genet.

[CR48] Haber DA, Buckler AJ, Glaser T (1990). An internal deletion within an 11p13 zinc finger gene contributes to the development of Wilms’ tumor. Cell.

[CR49] Hägglund A-C, Jones I, Carlsson L (2017). A novel mouse model of anterior segment dysgenesis (ASD): conditional deletion of Tsc1 disrupts ciliary body and iris development. Dis Model Mech.

[CR50] Han JC, Liu Q-R, Jones M (2008). Brain-derived neurotrophic factor and obesity in the WAGR syndrome. N Engl J Med.

[CR51] Han JC, Thurm A, Golden Williams C (2013). Association of brain-derived neurotrophic factor (BDNF) haploinsufficiency with lower adaptive behaviour and reduced cognitive functioning in WAGR/11p13 deletion syndrome. Cortex.

[CR52] Hanson I, Jordan T, van Heyningen V (1994) Aniridia. In: Molecular genetics of inherited eye disorders. CRC Press

[CR53] Hanson I, Churchill A, Love J (1999). Missense mutations in the most ancient residues of the PAX6 paired domain underlie a spectrum of human congenital eye malformations. Hum Mol Genet.

[CR54] Hart AW, Mella S, Mendrychowski J (2013). The developmental regulator Pax6 is essential for maintenance of islet cell function in the adult mouse pancreas. PLoS One.

[CR55] Hastie ND (1992). Dominant negative mutations in the Wilms tumour (WT1) gene cause Denys–Drash syndrome–proof that a tumour-suppressor gene plays a crucial role in normal genitourinary development. Hum Mol Genet.

[CR56] Hayashi S, Uehara DT, Tanimoto K (2017). Comprehensive investigation of CASK mutations and other genetic etiologies in 41 patients with intellectual disability and microcephaly with pontine and cerebellar hypoplasia (MICPCH). PLoS One.

[CR57] Hever AM, Williamson KA, van Heyningen V (2006). Developmental malformations of the eye: the role of PAX6, SOX2 and OTX2. Clin Genet.

[CR58] Hill RE, Favor J, Hogan BL (1991). Mouse small eye results from mutations in a paired-like homeobox-containing gene. Nature.

[CR59] Hingorani M, Williamson KA, Moore AT, van Heyningen V (2009). Detailed ophthalmologic evaluation of 43 individuals with PAX6 mutations. Invest Ophthalmol Vis Sci.

[CR170] Hsiao C-T, Liu Y-T, Liao Y-C (2017). Mutational analysis of ITPR1 in a Taiwanese cohort with cerebellar ataxias. PLoS ONE.

[CR60] Hu C, Malik V, Chang YK (2017). Coop-Seq analysis demonstrates that Sox2 evokes latent specificities in the DNA recognition by Pax6. J Mol Biol.

[CR61] Huettl R-E, Eckstein S, Stahl T (2016). Functional dissection of the Pax6 paired domain: roles in neural tube patterning and peripheral nervous system development. Dev Biol.

[CR62] Imaizumi M, Kuwabara T (1971). Development of the rat iris. Invest Ophthalmol.

[CR63] Isken O, Maquat LE (2007). Quality control of eukaryotic mRNA: safeguarding cells from abnormal mRNA function. Genes Dev.

[CR64] Ito YA, Footz TK, Berry FB (2009). Severe molecular defects of a novel FOXC1 W152G mutation result in aniridia. Invest Ophthalmol Vis Sci.

[CR65] Jensen AM (2005). Potential roles for BMP and Pax genes in the development of iris smooth muscle. Dev Dyn.

[CR66] Jiang Q-X, Thrower EC, Chester DW (2002). Three-dimensional structure of the type 1 inositol 1,4,5-trisphosphate receptor at 24 A resolution. EMBO J.

[CR67] Jordan T, Hanson I, Zaletayev D (1992). The human PAX6 gene is mutated in two patients with aniridia. Nat Genet.

[CR68] Jun S, Desplan C (1996). Cooperative interactions between paired domain and homeodomain. Development.

[CR69] Kerem E, Konstan MW, De Boeck K et al (2014) Ataluren for the treatment of nonsense-mutation cystic fibrosis: a randomised, double-blind, placebo-controlled phase 3 trial. 2:539–547. 10.1016/S2213-2600(14)70100-6 10.1016/S2213-2600(14)70100-6 PMC4154311 24836205

[CR70] Kernie SG, Liebl DJ, Parada LF (2000). BDNF regulates eating behavior and locomotor activity in mice. EMBO J.

[CR71] Kervestin S, Jacobson A (2012). NMD: a multifaceted response to premature translational termination. Nat Rev Mol Cell Biol.

[CR72] Kim J, Lauderdale JD (2006). Analysis of Pax6 expression using a BAC transgene reveals the presence of a paired-less isoform of Pax6 in the eye and olfactory bulb. Dev Biol.

[CR73] Klar J, Ali Z, Farooq M (2017). A missense variant in ITPR1 provides evidence for autosomal recessive SCA29 with asymptomatic cerebellar hypoplasia in carriers. Eur J Hum Genet.

[CR74] Kleinjan DA, Seawright A, Childs AJ, van Heyningen V (2004). Conserved elements in Pax6 intron 7 involved in (auto)regulation and alternative transcription. Dev Biol.

[CR75] Knudson AG (1971). Mutation and cancer: statistical study of retinoblastoma. Proc Natl Acad Sci USA.

[CR76] Koller A, Schlossmann J, Ashman K (2003). Association of phospholamban with a cGMP kinase signaling complex. Biochem Biophys Res Commun.

[CR77] Kondoh H, Uchikawa M, Kamachi Y (2004). Interplay of Pax6 and SOX2 in lens development as a paradigm of genetic switch mechanisms for cell differentiation. Int J Dev Biol.

[CR78] Kozmik Z, Czerny T, Busslinger M (1997). Alternatively spliced insertions in the paired domain restrict the DNA sequence specificity of Pax6 and Pax8. EMBO J.

[CR79] Lakowski J, Majumder A, Lauderdale JD (2007). Mechanisms controlling Pax6 isoform expression in the retina have been conserved between teleosts and mammals. Dev Biol.

[CR80] Lauderdale JD, Wilensky JS, Oliver ER (2000). 3′ deletions cause aniridia by preventing PAX6 gene expression. Proc Natl Acad Sci USA.

[CR81] Lek M, Karczewski KJ, Minikel EV (2016). Analysis of protein-coding genetic variation in 60,706 humans. Nature.

[CR82] Liu H, Xu S, Wang Y (2007). Ciliary margin transdifferentiation from neural retina is controlled by canonical Wnt signaling. Dev Biol.

[CR83] Lyons WE, Mamounas LA, Ricaurte GA (1999). Brain-derived neurotrophic factor-deficient mice develop aggressiveness and hyperphagia in conjunction with brain serotonergic abnormalities. Proc Natl Acad Sci USA.

[CR84] Maeda N, Niinobe M, Mikoshiba K (1990). A cerebellar Purkinje cell marker P400 protein is an inositol 1,4,5-trisphosphate (InsP3) receptor protein. Purification and characterization of InsP3 receptor complex. EMBO J.

[CR85] Mann IC (1925). The development of the human iris. Br J Ophthalmol.

[CR86] Mariën P, Brouns R, Engelborghs S (2008). Cerebellar cognitive affective syndrome without global mental retardation in two relatives with Gillespie syndrome. Cortex.

[CR87] Marlin S, Couet D, Lacombe D (1994). Obesity: a new feature of WAGR (del 11p) syndrome. Clin Dysmorphol.

[CR88] Mataftsi A (2016). Incidence of and risk factors for postoperative glaucoma and its treatment in paediatric cataract surgery. Dev Ophthalmol.

[CR89] Matsumoto M, Nakagawa T, Inoue T (1996). Ataxia and epileptic seizures in mice lacking type 1 inositol 1,4,5-trisphosphate receptor. Nature.

[CR90] Matsuo T, Osumi-Yamashita N, Noji S (1993). A mutation in the Pax-6 gene in rat small eye is associated with impaired migration of midbrain crest cells. Nat Genet.

[CR91] Maurya SK, Mishra R (2017). Pax6 binds to promoter sequence elements associated with immunological surveillance and energy homeostasis in brain of aging mice. Ann Neurosci.

[CR92] Mayer KL, Nordlund ML, Schwartz GS, Holland EJ (2003). Keratopathy in congenital aniridia. Ocul Surf.

[CR93] McDonald CM, Campbell C, Torricelli RE (2017). Ataluren in patients with nonsense mutation Duchenne muscular dystrophy (ACT DMD): a multicentre, randomised, double-blind, placebo-controlled, phase 3 trial. Lancet.

[CR94] McEntagart M, Williamson KA, Rainger JK (2016). A Restricted repertoire of de novo mutations in ITPR1 cause gillespie syndrome with evidence for dominant-negative effect. Am J Hum Genet.

[CR95] Menke A, Casagrande S, Geiss L, Cowie CC (2015). Prevalence of and trends in diabetes among adults in the United States, 1988–2012. JAMA.

[CR96] Miller RW, Fraumeni JF, Manning MD (1964). Association of Wilms’s tumor with aniridia, hemihypertrophy and other congenital malformations. N Engl J Med.

[CR97] Mishra R, Gorlov IP, Chao LY (2002). PAX6, paired domain influences sequence recognition by the homeodomain. J Biol Chem.

[CR98] Mitchell TN, Free SL, Williamson KA (2003). Polymicrogyria and absence of pineal gland due to PAX6 mutation. Ann Neurol.

[CR99] Moreland JL, Gramada A, Buzko OV (2005). The Molecular Biology Toolkit (MBT): a modular platform for developing molecular visualization applications. BMC Bioinform.

[CR100] Nallathambi J, Neethirajan G, Shashikant S (2006). PAX6 missense mutations associated in patients with optic nerve malformation. Mol Vis.

[CR101] Nanjo Y, Kawasaki S, Mori K (2004). A novel mutation in the alternative splice region of the PAX6 gene in a patient with Peters’ anomaly. Br J Ophthalmol.

[CR102] Natan E, Wells JN, Teichmann SA, Marsh JA (2016). Regulation, evolution and consequences of cotranslational protein complex assembly. Curr Opin Struct Biol.

[CR103] Nelson LB, Spaeth GL, Nowinski TS (1984). Aniridia. A review. Surv Ophthalmol.

[CR104] Netland PA (2015). Management of glaucoma in congenital aniridia. Aniridia.

[CR105] Netland PA, Scott ML, Boyle JW, Lauderdale JD (2011). Ocular and systemic findings in a survey of aniridia subjects. J AAPOS.

[CR106] Neveu MM, Holder GE, Sloper JJ, Jeffery G (2005). Optic chiasm formation in humans is independent of foveal development. Eur J Neurosci.

[CR169] Nischal KK (2015). Genetics of congenital corneal opacification—impact on diagnosis and treatment. Cornea.

[CR107] Ogura H, Matsumoto M, Mikoshiba K (2001). Motor discoordination in mutant mice heterozygous for the type 1 inositol 1,4,5-trisphosphate receptor. Behav Brain Res.

[CR108] Parolin Schnekenberg R, Perkins EM, Miller JW (2015). De novo point mutations in patients diagnosed with ataxic cerebral palsy. Brain.

[CR109] Patel S, Joseph SK, Thomas AP (1999). Molecular properties of inositol 1,4,5-trisphosphate receptors. Cell Calcium.

[CR110] Pelletier J, Bruening W, Kashtan CE (1991). Germline mutations in the Wilms’ tumor suppressor gene are associated with abnormal urogenital development in Denys–Drash syndrome. Cell.

[CR111] Perveen R, Lloyd IC, Clayton-Smith J (2000). Phenotypic variability and asymmetry of Rieger syndrome associated with PITX2 mutations. Invest Ophthalmol Vis Sci.

[CR112] Ploski JE, Shamsher MK, Radu A (2004). Paired-type homeodomain transcription factors are imported into the nucleus by Karyopherin 13. Mol Cell Biol.

[CR113] Pritchard-Jones K, Graf N, van Tinteren H, Craft A (2016). Evidence for a delay in diagnosis of Wilms’ tumour in the UK compared with Germany: implications for primary care for children. Arch Dis Child.

[CR114] Prokudin I, Simons C, Grigg JR (2014). Exome sequencing in developmental eye disease leads to identification of causal variants in GJA8, CRYGC, PAX6 and CYP1B1. Eur J Hum Genet.

[CR115] Prosser J, van Heyningen V (1998). PAX6 mutations reviewed. Hum Mutat.

[CR116] Prota G, Hu DN, Vincensi MR (1998). Characterization of melanins in human irides and cultured uveal melanocytes from eyes of different colors. Exp Eye Res.

[CR117] Regalado ES, Mellor-Crummey L, De Backer J (2018). Clinical history and management recommendations of the smooth muscle dysfunction syndrome due to ACTA2 arginine 179 alterations. Genet Med.

[CR118] Rennie IG (2012). Don’t it make my blue eyes brown: heterochromia and other abnormalities of the iris. Eye (Lond).

[CR119] Riccardi VM, Sujansky E, Smith AC, Francke U (1978). Chromosomal imbalance in the Aniridia-Wilms’ tumor association: 11p interstitial deletion. Pediatrics.

[CR120] Roberts RC (1967). Small eyes—a new dominant eye mutant in the mouse. Genet Res.

[CR121] Robinson DO, Howarth RJ, Williamson KA (2008). Genetic analysis of chromosome 11p13 and the PAX6 gene in a series of 125 cases referred with aniridia. Am J Med Genet A.

[CR122] Rodríguez-López R, Pérez JMC, Balsera AM (2013). The modifier effect of the BDNF gene in the phenotype of the WAGRO syndrome. Gene.

[CR153] van Rossum DB, Patterson RL, Cheung K-H (2006). DANGER, a novel regulatory protein of inositol 1,4,5-trisphosphate-receptor activity. J Biol Chem.

[CR123] Roulez FMJ, Faes F, Delbeke P (2014). Congenital fixed dilated pupils due to ACTA2-multisystemic smooth muscle dysfunction syndrome. J Neuroophthalmol.

[CR124] Sadagopan KA, Liu GT, Capasso JE (2015). Anirdia-like phenotype caused by 6p25 dosage aberrations. Am J Med Genet A.

[CR125] Sale MM, Craig JE, Charlesworth JC (2002). Broad phenotypic variability in a single pedigree with a novel 1410delC mutation in the PST domain of the PAX6 gene. Hum Mutat.

[CR126] Schedl A, Ross A, Lee M (1996). Influence of PAX6 gene dosage on development: overexpression causes severe eye abnormalities. Cell.

[CR127] Schilter KF, Reis LM, Schneider A (2013). Whole-genome copy number variation analysis in anophthalmia and microphthalmia. Clin Genet.

[CR128] Schmidt-Sidor B, Szymańska K, Williamson K (2009). Malformations of the brain in two fetuses with a compound heterozygosity for two PAX6 mutations. Folia Neuropathol.

[CR129] Schneider S, Osher RH, Burk SE (2003). Thinning of the anterior capsule associated with congenital aniridia. J Cataract Refract Surg.

[CR130] Schwarz M, Cecconi F, Bernier G (2000). Spatial specification of mammalian eye territories by reciprocal transcriptional repression of Pax2 and Pax6. Development.

[CR131] Shaw MW, Falls HF, Neel JV (1960). Congenital aniridia. Am J Hum Genet.

[CR132] Shin DH, Kwon BS, Chang YP (2003). Ultramicroscopical immunolocalization of PAX6 in the adult chicken retina. Acta Histochem.

[CR133] Shinawi M, Sahoo T, Maranda B (2011). 11p14.1 microdeletions associated with ADHD, autism, developmental delay, and obesity. Am J Med Genet A.

[CR134] Simola KO, Knuutila S, Kaitila I (1983). Familial aniridia and translocation t(4;11)(q22;p13) without Wilms’ tumor. Hum Genet.

[CR135] Simpson TI, Price DJ (2002). Pax6; a pleiotropic player in development. Bioessays.

[CR136] Singh S, Chao LY, Mishra R (2001). Missense mutation at the C-terminus of PAX6 negatively modulates homeodomain function. Hum Mol Genet.

[CR137] Singh S, Mishra R, Arango NA (2002). Iris hypoplasia in mice that lack the alternatively spliced Pax6(5a) isoform. Proc Natl Acad Sci USA.

[CR138] Sisodiya SM, Free SL, Williamson KA (2001). PAX6 haploinsufficiency causes cerebral malformation and olfactory dysfunction in humans. Nat Genet.

[CR139] Sivak JM, West-Mays JA, Yee A (2003). Transcription factors Pax6 and AP-2 interact to coordinate corneal epithelial repair by controlling expression of matrix metalloproteinase gelatinase B. Mol Cell Biol.

[CR140] Solomon BD, Pineda-Alvarez DE, Balog JZ (2009). Compound heterozygosity for mutations in PAX6 in a patient with complex brain anomaly, neonatal diabetes mellitus, and microophthalmia. Am J Med Genet A.

[CR141] Sturm RA, Larsson M (2009). Genetics of human iris colour and patterns. Pigment Cell Melanoma Res.

[CR142] Sun J, Rockowitz S, Xie Q (2015). Identification of in vivo DNA-binding mechanisms of Pax6 and reconstruction of Pax6-dependent gene regulatory networks during forebrain and lens development. Nucleic Acids Res.

[CR143] Swisa A, Avrahami D, Eden N (2017). PAX6 maintains β cell identity by repressing genes of alternative islet cell types. J Clin Invest.

[CR144] Tabata H, Koinui A, Ogura A et al (2018) A novel nuclear localization signal spans the linker of the two DNA-binding subdomains in the conserved paired domain of Pax6. Genes Genet Syst 17–00057. 10.1266/ggs.17-00057 10.1266/ggs.17-00057 29607880

[CR145] Tada M, Nishizawa M, Onodera O (2016). Roles of inositol 1,4,5-trisphosphate receptors in spinocerebellar ataxias. Neurochem Int.

[CR146] The UniProt Consortium (2017). UniProt: the universal protein knowledgebase. Nucleic Acids Res.

[CR147] Tiberio G, Digilio MC, Giannotti A (2000). Obesity and WAGR syndrome. Clin Dysmorphol.

[CR148] Ticho BH, Hilchie-Schmidt C, Egel RT (2006). Ocular findings in Gillespie-like syndrome: association with a new PAX6 mutation. Ophthalmic Genet.

[CR149] Ton CC, Hirvonen H, Miwa H (1991). Positional cloning and characterization of a paired box- and homeobox-containing gene from the aniridia region. Cell.

[CR150] Tripathi R, Mishra R (2010). Interaction of Pax6 with SPARC and p53 in brain of mice indicates Smad3 dependent auto-regulation. J Mol Neurosci.

[CR151] Tzoulaki I, White IMS, Hanson IM (2005). PAX6 mutations: genotype-phenotype correlations. BMC Genet.

[CR152] Valencia CA, Husami A, Holle J (2015). Clinical impact and cost-effectiveness of whole exome sequencing as a diagnostic tool: a pediatric center’s experience. Front Pediatr.

[CR154] van Dijk T, Barth P, Reneman L (2017). A de novo missense mutation in the inositol 1,4,5-triphosphate receptor type 1 gene causing severe pontine and cerebellar hypoplasia: expanding the phenotype of ITPR1-related spinocerebellar ataxia’s. Am J Med Genet A.

[CR155] van der Meer-de Jong R, Dickinson ME, Woychik RP (1990). Location of the gene involving the small eye mutation on mouse chromosome 2 suggests homology with human aniridia 2 (AN2). Genomics.

[CR156] Walther C, Guenet JL, Simon D (1991). Pax: a murine multigene family of paired box-containing genes. Genomics.

[CR157] Wang X, Gregory-Evans CY (2015). Nonsense suppression therapies in ocular genetic diseases. Cell Mol Life Sci.

[CR158] Wang X, Shan X, Gregory-Evans CY (2016). A mouse model of aniridia reveals the in vivo downstream targets of Pax6 driving iris and ciliary body development in the eye. Biochim Biophys Acta.

[CR159] Wen W, Cho Y-S, Zheng W (2012). Meta-analysis identifies common variants associated with body mass index in east Asians. Nat Genet.

[CR160] Williams AL, Bohnsack BL (2015). Neural crest derivatives in ocular development: discerning the eye of the storm. Birth Defect Res C.

[CR161] Williamson KA, FitzPatrick DR (2014). The genetic architecture of microphthalmia, anophthalmia and coloboma. Eur J Med Genet.

[CR162] Xu HE, Rould MA, Xu W (1999). Crystal structure of the human Pax6 paired domain-DNA complex reveals specific roles for the linker region and carboxy-terminal subdomain in DNA binding. Genes Dev.

[CR163] Xu S, Han JC, Morales A (2008). Characterization of 11p14–p12 deletion in WAGR syndrome by array CGH for identifying genes contributing to mental retardation and autism. Cytogenet Genome Res.

[CR164] Yasuda T, Kajimoto Y, Fujitani Y (2002). PAX6 mutation as a genetic factor common to aniridia and glucose intolerance. Diabetes.

[CR165] Yogarajah M, Matarin M, Vollmar C (2016). PAX6, brain structure and function in human adults: advanced MRI in aniridia. Ann Clin Transl Neurol.

[CR166] Zambonin JL, Bellomo A, Ben-Pazi H (2017). Spinocerebellar ataxia type 29 due to mutations in ITPR1: a case series and review of this emerging congenital ataxia. Orphanet J Rare Dis.

[CR167] Zhang Y, Ferreira HB, Greenstein D (1998). Regulated nuclear entry of the *C. elegans* Pax-6 transcription factor. Mech Dev.

[CR168] Zhang X, Qin G, Chen G (2015). Variants in TRIM44 cause aniridia by impairing PAX6 expression. Hum Mutat.

